# Supplementary material for: Exploring Patients’ Perspectives on Late Complications after Colorectal and Anal Cancer Treatment: A Qualitative Study
Source: Curr Oncol. 2023 Aug 10;30(8):7532–41. doi: 10.3390/curroncol30080546 (PMC10453413; doi:10.3390/curroncol30080546)
Supplement: Supplementary file 1 [file curroncol-30-00546-s001.zip › curroncol-2507155-SI.pdf]

File S1. Interview guide about symptoms after treatment for colorectal and anal cancer

Research questions	Subheadings	Concrete questions
**Introduction, where the participant is allowed to speak freely for a while. Open questions and possibly clarification about consent and the course of the conversation. Information is also provided here about the study, confidentiality, and consent.**	Demographics and background	Name, age, reason for contacting the late complication clinic, number of contacts (phone or in-person)
		Inform that we have confidentiality obligations, and names and social security numbers (CPR numbers) are never entered
**Transition from cancer patient to cancer survivor**		Can you describe the difference between being a cancer patient and now a cancer survivor?<br><br>In relation to the activities you were engaged in before your cancer diagnosis (e.g., work, education, leisure), to what extent does your current life resemble your previous one?<br><br>Do you feel that healthcare professionals treat you differently as a cancer survivor?<br><br>Do you experience a daily fear of recurrence?<br><br>Do you consider yourself a "late complication patient"?
**Action strategies**	Personal strategies / new and better strategies	Before coming to the late complication clinic, did you have specific strategies for coping with your late effects in everyday life?<br><br>Why did you start treating your symptoms on your own?<br><br>Who could you have contacted for help?<br><br>How did self-treatment work for you?<br><br>Have the strategies changed after contacting the late complication clinic?
**Changes for you personally**	Effect on a personal level	Has the contact with the late complication clinic had an impact on you? Do you feel better, unchanged, or worse than before?<br><br>Do you feel that you have someone to turn to when you have questions, and does it provide you with a sense of security, or does it make no difference to you?
